# Effect of Neck Rotation With Flexion on Ultrasonographic Optic Nerve Sheath Diameter in Patients Undergoing Elective Craniotomy

**DOI:** 10.7759/cureus.55760

**Published:** 2024-03-07

**Authors:** Kandukuri Shiva Priya, Ashutosh Kaushal, Anuj Jain, Harish Kumar, Pranita Mandal, Vaishali Waindeskar, Rhea Thotungal, Sweta Kumari, Sunaina T Karna, Ujjwal Gupta

**Affiliations:** 1 Neuroanesthesiology and Critical Care, All India Institute of Medical Sciences, New Delhi, New Delhi, IND; 2 Anesthesiology, All India Institute of Medical Sciences, Bhopal, Bhopal, IND; 3 Microbiology, All India Institute of Medical Sciences, Bhopal, Bhopal, IND

**Keywords:** emergence time, craniotomy, surgical position, neck flexion, neck rotation, ultrasonography (usg), onsd = optic nerve sheath diameter

## Abstract

Background

Extreme neck positioning to facilitate craniotomy can result in impaired venous drainage from the brain and a subsequent rise in increased intracranial pressure (ICP). The effects of varied neck positioning intraoperatively on ultrasonographic optic nerve sheath diameter (USG-ONSD) are still unexplored. This study aims to quantify the angle of neck rotation and flexion that can cause a significant increase in USG-ONSD in patients undergoing elective craniotomy.

Methods

A total of 100 patients were recruited in this non-randomized study and equally divided into two groups. In one group, patients with neck rotation ≤30 degrees and in another group, patients with neck rotation >30 degrees with varying degrees of neck flexion were included. The average of three USG-ONSD measurements in both eyes was obtained and compared in both groups at baseline, after positioning, and at the end of the surgery after making the neck neutral.

Results

The results of 100 recruited patients were analyzed. All the patients had neck flexion in the range of 40° to 45°, whereas the neck rotation ranged from 10° to 45°. The USG-ONSD of both eyes changed significantly from baseline to post-positioning time point in patients with neck rotation >30° (right eye p=0.038, left eye p=0.04) when compared to neck rotation ≤30°. There was no significant change in USG-ONSD from baseline to the postoperative time point after making the neck neutral (right eye p=0.245, left eye p=0.850) in both groups.

Conclusions

This study demonstrates that USG-ONSD, a surrogate measure of ICP, increased significantly after neck flexion with rotation >30° in neurosurgical patients. However, USG-ONSD becomes comparable to baseline after putting the patient's neck in a neutral position after surgery.

## Introduction

During neurosurgical procedures, it is common practice to adopt different positions (supine, prone, lateral, sitting) with different ranges of neck flexion, extension, and rotation [1]. Extreme positioning can result in impaired venous drainage from the brain and increased intracranial pressure (ICP) [2-4]. Hence, monitoring intracranial pressure directly or indirectly can help in the proper positioning of the patient.

Ultrasonographic optic nerve sheath diameter (USG-ONSD) measurement is a practical, inexpensive, convenient, and reliable type of indirect ICP measuring modality that helps in assessing ICP non-invasively [5,6]. Many research works have investigated the effect of Trendelenburg, reverse Trendelenburg, and prone positioning on USG-ONSD [7-10].

There is a literature deficit regarding the effect of the angle of neck rotation with flexion relative to a neutral position on the USG-ONSD value. This study intended to quantify the angle of rotation with neck flexion that can cause a significant increase in USG-ONSD value in patients undergoing elective craniotomy.

The primary objective of this study was to estimate and compare the USG-ONSD, whereas the secondary objective was to estimate and compare emergence time in patients undergoing elective craniotomy with ≤30 degrees and >30 degrees of neck rotation and varying degrees of neck flexion.

## Materials and methods

This prospective non-randomized study was conducted in one of the tertiary care centers after ethical clearance from the institutional ethical committee (IHEC-LOP/2022/IL040) and after registering prospectively under the Clinical Trials Registry of India (CTRI/2022/11/047071). An informed, written consent was obtained from all the patients included in the study.

Patients aged 18 to 65 years, belonging to the American Society of Anesthesiologists (ASA) physical status I and II scheduled for elective craniotomy requiring placement of a Mayfield skull clamp, were included. Whereas non-consenting patients with pre-existing pathological eye conditions (optic neuritis, ocular injuries, ocular diseases, a history of ophthalmic surgery), patients undergoing neurosurgery in head neutral position, prone position, and preoperative raised ICP, midline shift of more than 5 mm in preoperative computed tomography imaging were excluded from this study. Patients not extubated for surgical reasons have been excluded from the study.

A total of 100 patients were recruited and equally divided into two groups. In one group, patients with neck rotation ≤30 degrees, and in the other group, patients with neck rotation >30 degrees with varying degrees of neck flexion as per the surgeon's comfort and exposure were included. All the patients included in the study received standard ASA monitoring. General anesthesia was induced with 2 mg/kg of I.V. propofol titrated with the loss of verbal command. After achieving neuromuscular blockade with intravenous vecuronium 0.10 mg/kg, direct laryngoscopy and intubation of the trachea with an appropriate-size endotracheal tube were performed. Anesthesia was maintained with oxygen and nitrous oxide in a ratio of 40%:60% with isoflurane to achieve a minimum alveolar concentration of 0.5 to 1. Neuromuscular block was maintained with intermittent boluses of I.V. vecuronium. Intermittent positive pressure ventilation with a tidal volume of 6-8 ml/kg body weight was instituted to maintain end-tidal carbon dioxide between 30 and 35 mm Hg. The depth of anesthesia was adjusted to control the bispectral index value between 40 and 60. Normothermia and normocapnia were maintained throughout the procedure, and heart rate (HR) and mean arterial pressure (MAP) were maintained within 20% of baseline. Patients were extubated at the end of the procedure as per extubation criteria.

USG-ONSD measurements were obtained by a single investigator before induction with the head in a neutral position (baseline reading), 10 minutes after positioning of the head on the Mayfield skull clamp, and at the end of the surgery after placing the neck in a neutral position. At each time point, USG-ONSD is performed three times, and an average of the values has been taken to analyze the results. A significant change in USG-ONSD was considered when it exceeded 4.6 mm for females and 4.8 mm for males. Emergence time was defined as the time taken for the patient to regain consciousness from the time anesthetic supply had been stopped and was noted down.

The sample size for the study was based on a study in which investigators reported the proportion of people with a significant rise in ICP after head maneuvers as 50%. Considering the prevalence of target condition p=0.50, δ (precision)=0.1 (10%), type I error (α)=5%, and 95% confidence interval, the proposed sample size for the study obtained was 100.

**Figure 1:**
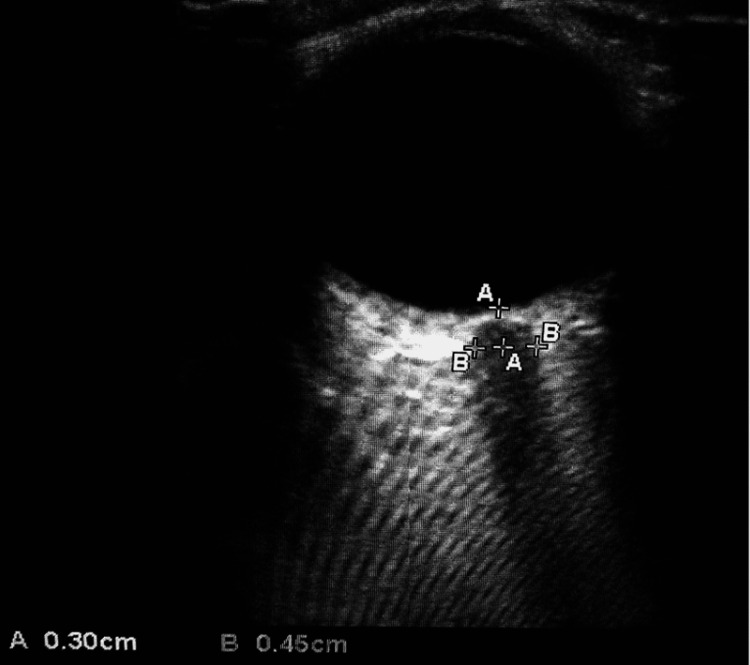
USG-ONSD measurement. A: 3 mm posterior to the posterior surface of the globe. B: Optic nerve sheath diameter. USG-ONSD: ultrasonographic optic nerve sheath diameter.

A 7.5 MHz linear transducer of USG (SonoSite M-Turbo; Fujifilm Corporation, COO-Japan) was placed over the closed eyelid in the transverse plane of each eye. The placement of the transducer was adjusted to bring the best angle for displaying the exit of the optic nerve from the globe. The widest visible retrobulbar ONSD was measured at a point 3 mm posterior to the posterior scleral surface of the globe using an in-built electronic caliper to the nearest millimeter, with an angle perpendicular to the eyeball. Figure 1 shows the USG-ONSD measurement.

Universal Goniometer (IS Indosurgicals, COO-India) was used to make measurements. The rotation axis (fulcrum) of the goniometer will be aligned to the most prominent center part of the head. The stationary arm was placed in line with the acromion process of the same side of rotation. The moving arm was placed in line with the tip of the nose. The flexion axis was aligned with the external auditory meatus. The stationary arm was placed vertically. The moving arm was placed in line with the nostrils.

The outcomes of this study were to evaluate whether more than 30 degrees of neck rotation with neck flexion will significantly increase USG-ONSD and emergence time when compared to ≤30 degrees of neck rotation with neck flexion in patients undergoing elective craniotomy.

Statistical Package for the Social Sciences version 23 (IBM Corp., Armonk, New York) was used for data analysis. Descriptive statistics were elaborated as means/standard deviations and medians/interquartile range (IQRs) for continuous variables and frequencies and percentages for categorical variables. Group comparisons for continuously distributed data were made using the independent sample ‘t’ test when comparing two groups and one-way ANOVA when comparing more than two groups. Post-hoc pairwise analysis was performed using Tukey’s honestly significant difference (HSD) test in the case of a one-way ANOVA to control for alpha inflation. A chi-squared test was used for group comparisons of categorical data. Statistical significance was kept at p < 0.05.

## Results

A total of 100 recruited patients were analyzed. Both groups of patients were comparable in terms of age, body mass index (BMI), neck circumference, and duration of surgery, as shown in Table 1. All the patients had neck flexion in the range of 40 to 45°, whereas the neck rotation ranged from 10° to 45°.

**Table 1:** Patient characteristics. SD: standard deviation, n: number, BMI: body mass index. p < 0.05 is considered significant.

Parameter		Neck rotation		p-value
		≤30° (n=50)	>30° (n=50)	
Age (years), mean ± SD		39.72 ± 14.19	39.07 ± 14.49	0.859
BMI (kg/m^2^), mean ± SD		25.67 ± 3.77	23.80 ± 4.67	0.090
Gender, n (%)	Male	42 (58.3%)	30 (41.7%)	0.144
	Female	10 (35.7%)	18 (64.3%)	
Neck circumference (cm), mean ± SD		36.87 ± 4.39	35.13 ± 4.02	0.06
Duration of surgery (hours), mean ± SD		4.62 ± 2.50	3.73 ± 1.62	0.152

The association between neck rotation and absolute USG-ONSD is shown in Table 2.

**Table 2:** Association between USG-ONSD and neck rotation. USG-ONSD: ultrasonographic optic nerve sheath diameter, SD: standard deviation, mm: millimeter, RE: right eye, LE: left eye. p < 0.05 is considered significant.

Time points	Absolute USG-ONSD (mm), mean ± SD		P-value
	Neck rotation ≤30° (n=50)	Neck rotation >30° (n=50)	
RE (baseline)	3.99 ± 0.51	3.79 ± 0.53	0.134
RE (after position)	4.34 ± 0.43	4.59 ± 0.47	0.038
RE (post-operative)	4.12 ± 0.44	3.98 ± 0.48	0.245
LE (baseline)	4.04 ± 0.44	3.90 ± 0.45	0.238
LE (after position)	4.30 ± 0.46	4.53 ± 0.50	0.040
LE (post-operative)	4.09 ± 0.39	4.08 ± 0.39	0.850

The USG-ONSD of both eyes changed significantly from baseline to post-positioning time point in patients with neck rotation >30° (right eye, p=0.038, left eye, p=0.04) when compared to neck rotation ≤30°. The emergence time was 14.71 ± 5.18 minutes and 13.68 ± 3.31 minutes in patients with ≤30° neck rotation and >30° neck rotation, respectively, which was comparable (p=0.758).

## Discussion

The objectives of this study were to estimate and compare the USG-ONSD value and emergence time in two groups of patients (≤30° versus >30° of neck rotation) undergoing craniotomy.

We found that the USG-ONSD of both eyes changed significantly from baseline to post-positioning time point in patients with neck rotation >30° when compared to neck rotation ≤30°, although the ONSD becomes insignificant when compared to baseline. In our study, USG-ONSD was considered to depict the change in ICP as it is a reliable, reproducible, and non-invasive modality for monitoring ICP. 

In a study conducted by Bauerle et al., ONSD in 40 healthy subjects, which was measured by two individuals, was assessed for intra- and inter-observer reliability, and they concluded that ONSD has high inter- and intra-observer reliability [11]. In the present study, ONSD was performed three times, and the mean of the values was taken by a single investigator in all the patients to address the drawback of inter-observer variability. 

One of the major factors that influences ICP is the venous drainage of the brain [1,12]. Venous drainage can be impeded during extreme positioning of the patient. Extreme neck flexion and rotation have been observed to be associated with impaired venous drainage and increased intracranial pressure [1,6,13].

Ample literature has been found on the effects of different postures on ONSD and ICP [2,6-9]. However, the effect of head rotation in any posture has not been commented on in any of the studies. Also, the optimum angle of head rotation or flexion that has significantly increased optical nerve sheath diameter has not been commented on.

Our study aimed to depict the changes of USG-ONSD in relation to the changes in the angle of rotation of the neck during positioning. In this study, the distance between the chin and sternum was assured to be less than two to three finger breadths in all the patients to avoid hyperflexion. We observed that a neck rotation of more than 30 degrees was associated with a significant change in ONSD after positioning.

In literature, it was stated that the head can typically be safely rotated up to 45 degrees away from the body. If more rotation is needed, a roll or pillow placement under the opposite shoulder is recommended [14]. However, in a recent review on surgical positioning for neurosurgical procedures, it is stated that the head shouldn’t be turned more than 30 degrees to one side, especially when turning it towards the dominant jugular vein [1]. In our study, a cut-off of a neck rotation angle of 30 degrees was taken for analysis.

The findings in our study that neck rotation of more than 30 degrees is associated with significant changes in ONSD are in concordance with the review on surgical positioning in neurosurgery, where head rotation of more than 30 degrees is stated to be detrimental [1]. Hence, it can be noted that ultrasonographic measurement of optic nerve sheath diameter can be used as an adjunctive tool along with clinical assessment to check proper positioning and avoid unwarranted situations like intraoperative tense brain.

There are a few strengths of this study. This is the first study to demonstrate the effect of different angles of neck rotation and neck flexion on USG-ONSD values in patients undergoing elective craniotomies. To avoid the possibility of inter-observer bias, all the USG-ONSD readings were taken by a single investigator. 

Our study has a few limitations. In our study, only patients with a midline shift of less than 5 mm were included. Hence, these findings cannot be extrapolated to the population with a raised ICP preoperatively. In this study, 75% of the patients included were male, and this could have been a confounding factor in the analysis of the absolute values of ONSD. Positions other than supine were not included. The angle of lateral flexion was not considered in these patients. Non-randomized study design and single-center study with a small sample size are other limitations. Also, postoperative follow-up for the clinical outcome was not done in these patients.

## Conclusions

This study demonstrates that USG-ONSD, a surrogate measure of ICP, increased significantly after neck flexion with rotation of more than 30 degrees in neurosurgical patients. However, USG-ONSD becomes insignificant after placing the patient's neck in a neutral position after surgery. In this study, we have observed that the changes in the cervical angle lead to a dynamic and significant change in USG-ONSD. Randomized study design with large sample size is needed to confirm the findings. 
